# 2363 Inducing anti-tumor immunity in colorectal cancer

**DOI:** 10.1017/cts.2018.87

**Published:** 2018-11-21

**Authors:** Jonathan B. Mitchem, Yue Guan, Mark Daniels, Emma Teixeiro

**Affiliations:** Institute of Clinical and Translational Sciences, Washington University in St. Louis, St. Louis, MO, USA

## Abstract

OBJECTIVES/SPECIFIC AIMS: Despite significant advances in screening and treatment, colorectal cancer is the second leading cancer killer in the United States today. Some of the most promising recent developments in cancer therapy have come from immune-based therapy. Immune-based therapy, however, has shown limited utility in patients with colorectal cancer. Studies have previously shown that certain chemotherapy regimens may be more effective in combination with immune-based therapy due to induction of inflammation in the tumor microenvironment. In this study, we sought to determine how standard chemotherapy (FOLFOX) affects the generation of antigen-specific anti-tumor immunity in colorectal cancer. METHODS/STUDY POPULATION: To determine the how antigen-specific immunity and T cell responses are affected by FOLFOX, we utilized a model antigen expressing murine colon cancer cell line syngeneic to C57BL/6 (MC38-CEA). Treatment was initiated when tumor size reached 50 mm^2^. Mice were treated with either vehicle (PBS), 5-Fluorouracil (5-FU), Oxaliplatin, or combination (FOLFOX). Antigen-specific cytotoxic T cell (tet+Tc) were detected using Db-CEA-tetramer obtained from the NIH-tetramer core facility. Flow cytometry was performed for phenotypic analysis and tetramer positivity. Tumor growth was measured using standard caliper measurements. Statistical analysis was performed using *t*-test for continuous variables and ANOVA was used when comparing multiple groups. Statistical analysis was performed using SPSS. All arms were completed with n=3–7. RESULTS/ANTICIPATED RESULTS: To determine how systemic treatment with chemotherapy affects cytotoxic T cell development (Tc), we established that we could detect antigen-specific Tc (tet+Tc) in the spleen, tumor, and draining lymph nodes of tumor-bearing mice. After establishing that the system worked appropriately, tumor-bearing mice were treated with different chemotherapy regimens and tumor growth was monitored. As expected, the combination of FOLFOX was significantly better than either drug individually (2-way ANOVA, *p*<0.01). FOLFOX therapy also showed a significant (*p*<0.05) increase in the number of tumor-associated tet+Tc, and tet+Tc expressing phenotypic markers of effector (Te) and resident memory (Trm) subsets. Tumor-associated tet+Tc highly expressed PD-1 (>50%); however, this was not significantly different between treatment or vehicle arms. Since 5-FU, one component of FOLFOX has previously shown a selective reduction of myeloid-derived suppressor cells, we also investigated the myeloid compartment. There were no significant differences in conventional or plasmacytoid dendritic cells, myeloid-derived suppressor cells, or tumor-associated macrophages. DISCUSSION/SIGNIFICANCE OF IMPACT: The future of cancer care involves multi-modality care tailored to patients. To more effectively combine therapy it is critical that we understand how currently utilized therapy works. In this study, we show that the primary chemotherapy regimen utilized in colorectal cancer increases tumor-associated antigen-specific cytotoxic T cells and the majority of these cells are PD-1 positive. This suggests that FOLFOX may work in concert with immune-based therapy when selected appropriately. Further study is warranted to determine optimal combination therapy and ways to maximize anti-tumor immunity in order to improve the treatment of patients with this deadly disease.

